# Characterization of a Peptide Domain within the GB Virus C NS5A Phosphoprotein that Inhibits HIV Replication

**DOI:** 10.1371/journal.pone.0002580

**Published:** 2008-07-02

**Authors:** Jinhua Xiang, James H. McLinden, Qing Chang, Emma L. Jordan, Jack T. Stapleton

**Affiliations:** Iowa City VA Medical Center and the University of Iowa, Iowa City, Iowa, United States of America; National Cancer Institute, United States of America

## Abstract

**Background:**

GBV-C infection is associated with prolonged survival in HIV-infected people and GBV-C inhibits HIV replication in co-infection models. Expression of the GBV-C nonstructural phosphoprotein 5A (NS5A) decreases surface levels of the HIV co-receptor CXCR4, induces the release of SDF-1 and inhibits HIV replication in Jurkat CD4+ T cell lines.

**Methodology/Principal Findings:**

Jurkat cell lines stably expressing NS5A protein and peptides were generated and HIV replication in these cell lines assessed. HIV replication was significantly inhibited in all cell lines expressing NS5A amino acids 152–165. Substitution of an either alanine or glycine for the serine at position 158 (S158A or S158G) resulted in a significant decrease in the HIV inhibitory effect. In contrast, substituting a phosphomimetic amino acid (glutamic acid; S158E) inhibited HIV as well as the parent peptide. HIV inhibition was associated with lower levels of surface expression of the HIV co-receptor CXCR4 and increased release of the CXCR4 ligand, SDF-1 compared to control cells. Incubation of CD4+ T cell lines with synthetic peptides containing amino acids 152–167 or the S158E mutant peptide prior to HIV infection resulted in HIV replication inhibition compared to control peptides.

**Conclusions/Significance:**

Expression of GBV-C NS5A amino acids 152–165 are sufficient to inhibit HIV replication *in vitro*, and the serine at position 158 appears important for this effect through either phosphorylation or structural changes in this peptide. The addition of synthetic peptides containing 152–167 or the S158E substitution to Jurkat cells resulted in HIV replication inhibition *in vitro*. These data suggest that GBV-C peptides or a peptide mimetic may offer a novel, cellular-based approach to antiretroviral therapy.

## Introduction

Unlike most *in vitro* systems developed to study HIV replication, humans infected with HIV are co-infected with a variety of pathogenic and nonpathogenic microbes. These coinfections may have unilateral or bidirectional interactions with HIV that may alter the clinical outcome of either infection. For example, HIV infection accelerates the course of hepatitis C virus (HCV) related hepatic disease (reviewed in [1]), and is associated with an increased prevalence of Kaposi's sarcoma in people with coexistent herpesvirus 8 (HHV-8) infection [2]. In contrast, persistent infection with GB virus type C (GBV-C) is associated with prolonged survival in HIV-infected individuals (reviewed in [3] and [4]). Identification of the mechanism(s) by which microbial coinfections inhibit HIV replication may identify novel therapeutic targets or assist in the development of prevention strategies. Microbial interactions may be direct, (e.g. the HIV tat protein stimulates HHV-8 replication)[5] or indirect (γHV68 stimulates prolonged production of the IFNγ, upregulating the basal state of innate immunity against subsequent infection)[6]. Several viral infections inhibit HIV replication *in vitro* including HHV-6 [7], HHV-7 [8], GBV-C [9]–[11], measles virus [12], [13], and vaccina virus [14]. Several of these infections alter HIV replication by decreasing expression of HIV coreceptors and inducing chemokines that compete with HIV for binding to entry receptors [10].

GBV-C is a common lymphotropic virus that may persist in infected humans for decades, although the majority of immune competent individuals clear viremia within two years of infection (reviewed in [15]). Antibody to the envelope glycoprotein E2 is usually detected following clearance of viremia, and detection of this antibody is indicative of prior GBV-C infection (reviewed in [1]). Viremia is present in approximately 2% of healthy blood donors in the United States [16], [17] and E2 antibodies are found in another 9% to 12% of these donors [18]–[22]. GBV-C prevalence is higher in developing countries and in U.S. populations that have other blood-born or sexually transmitted infections [15]. For example, approximately 20% of HCV-infected and up to 42% of HIV-infected individuals are viremic in cross-sectional studies (reviewed in [15]). Although GBV-C was initially thought to cause hepatitis, thorough investigation has failed to conclusively identify an association between the virus and hepatitis, or any other human disease [23].

On the basis of nucleotide sequence and predicted genome organization, GBV-C is classified in the family *Flaviviridae* [16], [17]. The virus replicates in human peripheral blood mononuclear cells (PBMCs) including B and T lymphocytes (CD4+ and CD8+ subsets)[24]–[28] and co-infection of PBMCs with GBV-C and HIV results in inhibition of HIV replication [9]–[11], [29], [30]. HIV inhibition is mediated in part by downregulation of the HIV co-receptors CCR5 and CXCR4 and by the induction of the ligands for these chemokine receptors (RANTES, MIP-1α, MIP-1β, SDF-1)[10], [11], [29], [30]. Consequently, GBV-C co-infection of PBMCs inhibits the replication of both CCR5- and CXCR4-tropic HIV isolates (R5 and X4 respectively), and HIV isolates representing clades A through H and the more divergent group O isolates [10], [11].

GBV-C has a single strand, positive sense RNA genome encoding a polyprotein of approximately 3000 amino acids [31]. GBV-C structural proteins reside at the amino terminal one third of the polyprotein and are processed by cellular signal peptidases, while the nonstructural proteins are processed by viral proteases [32]. Two GBV-C proteins have been shown to inhibit HIV replication *in vitro* [30], [33]. Addition of the envelope glycoprotein (E2) to CD4+ T cells inhibits R5 and X4 HIV isolates [30], [34]. Infection with GBV-C and exposure of cells to E2 protein decreased surface expression of the HIV entry coreceptor CCR5, and also induced RANTES, one of the three known ligands for CCR5. The mechanism by which E2 inhibits X4 viruses has not been fully characterized [30]. In addition to E2, expression of the GBV-C nonstructural phosphoprotein NS5A in a CD4+ T cell line (Jurkat) potently inhibits HIV replication [33]. GBV-C NS5A decreases surface expression of CXCR4, and increases the release of SDF-1 (the CXCR4 ligand) into cell culture supernatants [33], [35]. In this study, we further characterize the GBV-C NS5A peptide requirements involved in HIV inhibition by mutagenesis, and determined that synthetic peptides are able to reproduce the effects of the intracellularly expressed NS5A peptides.

## Results

### Characterization of the GBV-C NS5A domain required for HIV inhibition

**Figure 1:**
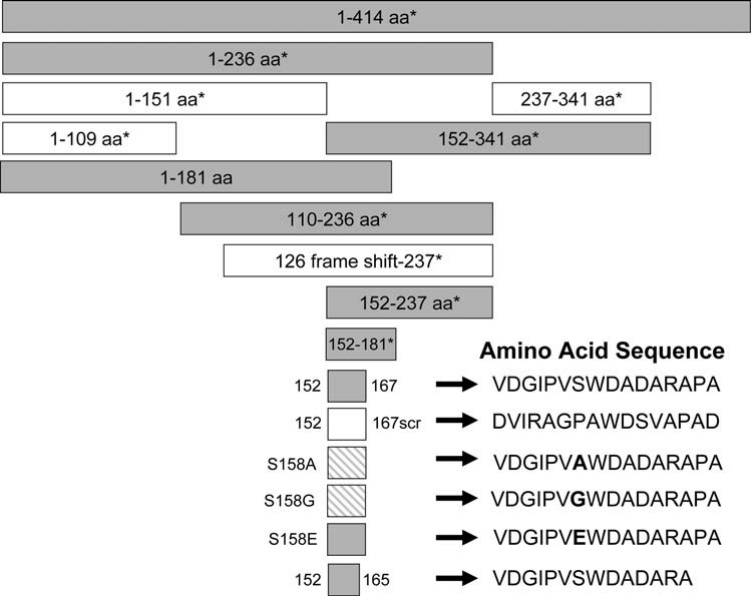
Summary of GBV-C NS5A proteins stably expressed in Jurkat – Tet-off cells. Boxes represent the amino acid region of GBV-C NS5A expressed in cloned Jurkat cell lines. Full length NS5A is 414 amino acids in length (1–414). The 126 frame-shift-237 contains GBV-C NS5A sequences that normally encodes these amino acids in the NS5A protein; however, a plus 1 frame shift was introduced so that the cells encode 26 missense amino acids. The amino acid sequence is shown for the 16mer peptide (152–167) and mutant peptides. Shaded boxes indicate cell lines in which HIV replication was inhibited compared to vector controls. Boxes with cross-hatches represent cell lines that inhibited HIV replication compared to controls, but significantly less than those cells indicated by solid shading. * = cell lines described previously.

**Figure 2:**
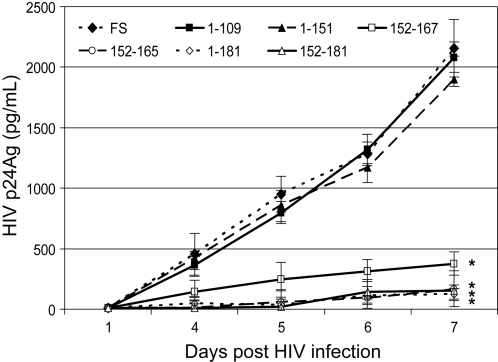
HIV growth characteristics in selected Jurkat cell lines expressing the GBV-C NS5A sequences. HIV replication in selected cell lines expressing various GBV-C NS5A peptides summarized in Fig. 1 was assessed by monitoring HIV p24 antigen release into culture supernatants. FS = the 126–237 frame-shift construct (see methods). Numbers refer to the NS5A amino acid sequence expressed in each cell line. * = HIV replication curves significantly less HIV p24 antigen than the frame shift control. (P<0.01 for the three cell lines that included amino acids 152–165 on days 3, 4, 5).

Previous studies demonstrated that stable expression of the GBV-C NS5A protein in a CD4+ T lymphocyte cell line (Jurkat cells) inhibited HIV replication [33]. The effect required protein expression, as cells expressing GBV-C NS5A RNA in which a frame-shift mutation that did not express NS5A did not inhibit HIV [33]. Based on the expression of peptide deletions of NS5A, the HIV inhibitory effect was mapped by sequential deletion mutational analysis to amino acids 152–181 [33], [35]. To further characterize the region within this 30 amino acid NS5A sequence responsible for HIV inhibition, Jurkat cell lines were generated that stably expressed a number of NS5A peptides, including some designed to mutate a specific amino acid. Representative NS5A fragments previously described and seven new NS5A constructs were used to generate stably expressing Jurkat cell lines in this study as summarized in Fig. 1. The NS5A sequences were inserted into a bicistronic vector that utilizes a tet-responsive CMV promoter to drive transcription, and for which the GBV-C NS5A sequences were expressed as the upstream open reading frame (ORF) followed by stop codons, and an encephalomyocarditis internal ribosomal entry site that directs translation of green fluorescent protein (GFP) from the downstream ORF as previously described [33]. HIV replication was significantly reduced in cell lines expressing GBV-C NS5A amino acids 1–181, 152–181, 152–167 and 152–165 when compared to HIV replication in three cell lines containing the NS5A frame-shift RNA and cells expressing NS5A amino acids 1–109 and 1–151 (Fig. 2). Thus all Jurkat cell lines that expressed GBV-C NS5A peptides containing amino acids 152–165 significantly inhibited HIV replication compared to the vector and frame-shift controls. Cell lines that included GBV-C NS5A coding regions that did not include NS5A amino acids 152–165 did not inhibit HIV (Fig. 2 and data not shown). Addition of doxycycline to decrease GBV-C NS5A peptide expression decreased the extent of HIV replication consistent with previous experiments [33](data not shown). Thus expression of a 14 amino acid region within the GBV-C NS5A protein (152–165) in this CD4+ T cell line was sufficient to significantly inhibit HIV replication. All HIV infections were performed in triplicate and repeated at least twice with consistent results.

**Figure 3:**
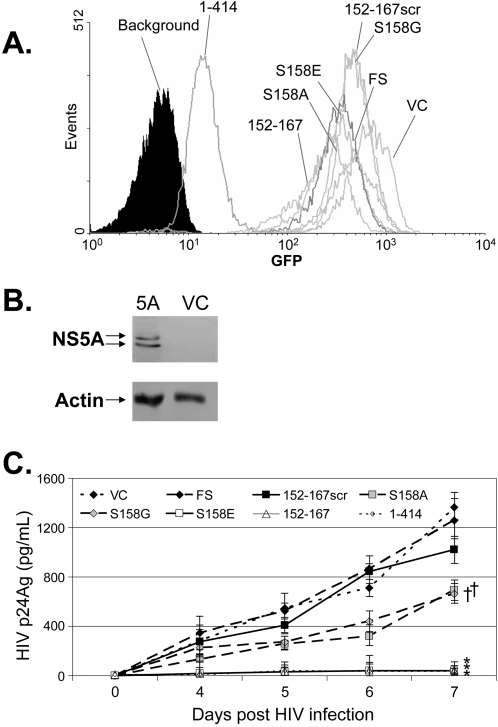
Mutational analysis of GBV-C 152–167 NS5A peptide. Clonal cell lines stably transfected with NS5A peptides were selected by hygromycin resistance and examined for GFP expression (A). Cells expressed the full length NS5A protein (amino acids 1–414), an NS5A 16 amino acid protein (152–167), the same 16 amino acids scrambled (152–167scr), or peptides in which the serine (position 158) was replaced with alanine (S158A), glycine (S158G) or glutamic acid (S158E). VC = vector control cells and FS = frame-shift control cells. Background represents the background fluorescence of parental Jurkat cells. Panel B demonstrates two immunoreactive NS5A proteins in cells expressing 1–414 (5A) but not in the vector control (VC) cells. Actin loading controls are shown. HIV replication was significantly decreased in Jurkat cell lines expressing full-length NS5A (1–414), the 152–167 peptide, and the S158E mutant (*) compared to all other cell lines on days 5, 6, and 7 days post-infection (panel C). The S158A and S158G cell lines had significantly less HIV replication than FS and VC (†; P = 0.041 on day 7), but significantly more HIV replication than the 1–414, 152–167, and S158E cells (†; P<0.02 for all, day 7).

The GBV-C NS5A protein is predicted to be a phosphoprotein, and expression of NS5A in cells results in two immunoreactive proteins consistent with a basal and hyperphosphorylated state [33], [36]. The 152–167 NS5A peptide contains a serine residue at position 158 that is predicted to be phosphorylated (http://www.cbs.dtu.dk/services/NetPhos/). To assess whether this serine residue is important for the HIV inhibitory effect, cell lines were generated that expressed the NS5A 16mer 152–167 (VDGIPVSWDADARAPA) or mutated NS5A 16mer peptides with nonconservative substitutions (S158A and S158G) or a phosphomimetic substitution (S158E) [37]. A negative control cell line was also generated that expressed the 152–167 amino acid sequence in a scrambled order (DVIRAGPAWDSVAPAD) that is not predicted to be phosphorylated (summarized in Fig. 1). Clonal cell lines for each construct were selected that expressed the GFP reporter gene (Fig. 3A). The relative expression of GFP varied between cell lines, consistent with previous studies showing that the larger the protein encoded in the upstream open reading frame in a bicistronic vector, the lower the expression of the IRES-driven downstream protein, in this case GFP [38]. Fig. 3B demonstrates that the full-length NS5A, which had the lowest level of GFP expression, expressed two immunoreactive proteins as shown previously [36].

Because the anti-NS5A antibody used in Fig. 3B does not detect the 16 amino acid NS5A peptides, additional testing was performed on cell lines containing the short peptide sequences. First, cellular DNA from the Jurkat cell lines was amplified using PCR primers upstream of the CMV promoter region and in the downstream GFP coding region, and the product including the entire GBV-C NS5A peptide coding region was sequenced as previously described [33]. For all of the cell lines shown in figure 3A, the NS5A peptide sequence was shown to be intact and linked to both IRES and GFP sequences [33], [35](data not shown). Secondly, total cellular RNA from the cells containing the NS5A peptides was extracted from each cell line, amplified by RT-PCR and the amplified products sequenced. All of these cell lines contained the NS5A mRNA linked to the CMV promoter and the downstream GFP sequences. No sequences were amplified when reverse transcriptase was not included in the buffer, thus confirming that the GBV-C peptide RNA sequences were transcribed in these cells (data not shown).

HIV replication was significantly inhibited in the cell lines expressing the S158E peptide compared to vector control, frame-shift control, S158A and S158G cell lines (Fig. 3C; p<0.01 for all; T-test on days 5, 6, and 7). Jurkat cells expressing S158A and S158G mutations also inhibited HIV replication compared to the vector, frame-shift, and 152–167 scrambled peptide controls (p = 0.037 day 6 and p = 0.043 day 7), but this inhibition was significantly less than that observed in cells expressing the S158E peptide (Fig. 3C; p = 0.014 day 6, p = 0.001 day 7). Thus although the serine residue at position 158 appears to be important for maximal HIV inhibition, mutation to nonconservative amino acids did not completely abrogate the inhibitory effect.

**Figure 4:**
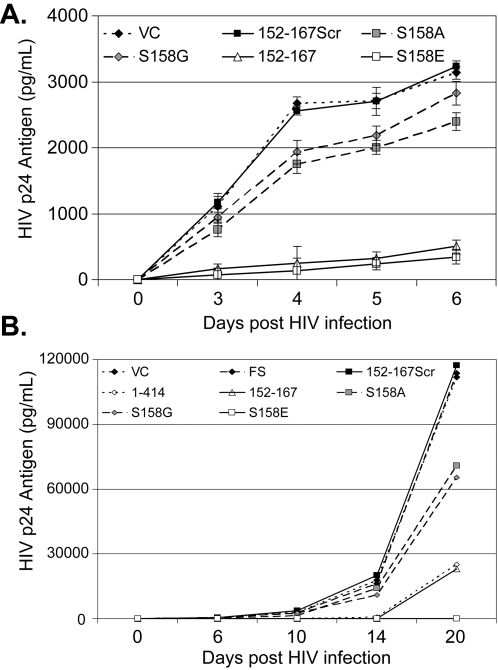
Expression of GBV-C NS5A peptides and HIV replication. To ensure that HIV inhibition was not due to abnormalities selected for during clonal selection of cell lines, HIV replication was assessed in the bulk cell lines that expressed the GBV-C NS5A 152–167 peptide, the scrambled peptide (152–167Scr), and the mutated peptides (S158A, S158G, S158E) and compared to the vector control cell line (VC) (panel A). The pattern of HIV inhibition did not change appreciably when infections were followed for up to 20 days (panel B). 1–414 = cells expressing the full-length GBV-C NS5A and FS = cells expressing the GBV-C NS5A frame shift control.

To ensure that these results were not the result of cellular abnormalities introduced during clonal selection, HIV replication in the bulk cell lines that were used to generate the clonal cell lines was analyzed. Figure 4A illustrates that HIV replication was significantly inhibited in these cell lines, and confirmed that expression of the GBV-C NS5A 152–167 peptide and the S158E peptide inhibited HIV significantly more than the vector control, scrambled peptide, and both S158G and S158A peptides (p<0.001 days 3 through 6). However, the S158G and S158A peptides were again shown to inhibit HIV replication when compared to the vector and scrambled peptide control cell lines. The pattern of HIV inhibition was not altered when HIV infection was allowed to proceed longer in the clonal Jurkat cell lines (up to 20 days; Fig. 4B).

Consistent with prior studies [33], expression of full-length and NS5A peptides did not have any morphologic effects on Jurkat cells nor did they alter viability. Specifically, cell number increased equally between vector control and NS5A-expressing cell lines after five days in culture, and >98% of cells were viable by MTT and trypan blue exclusion analyses for all cell lines (data not shown).

**Figure 5:**
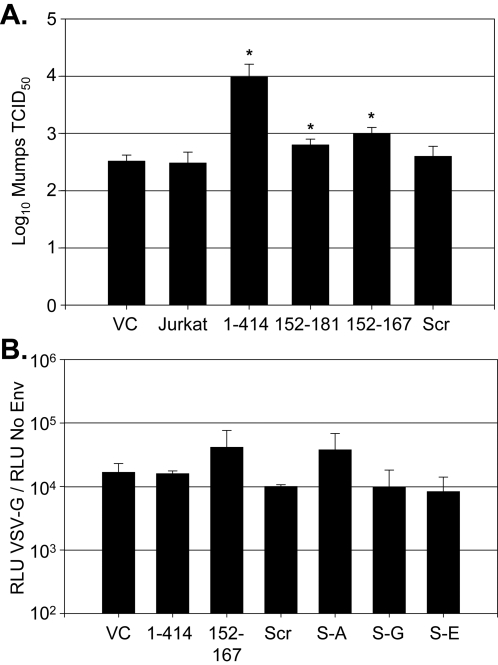
The inhibitory effect of NS5A expression is specific for HIV replication. The infectious titer of mumps virus was not inhibited in Jurkat cells expressing full-length GBV-C NS5A (1–414) or the 152–181 NS5A peptide compared to the vector control (VC) or parent Jurkat cells (A). Mumps virus replication was increased in Jurkat cells expressing full-length NS5A (*; p = 0.029). Similarly, panel B demonstrates that VSV-G pseudotyped retroviruses transduced all NS5A and control cell lines with similar efficiency as measured by relative luciferase activity in VSV-G pseudotype particles relative to the no-envelope control particles (p>0.05 for all compared to VC). 152–167 = cells expressing this NS5A 16mer peptide, Scr = cells expressing scrambled 16mer peptide, and S-A, S-G, S-E = cells expressing the S158A, S158G, and S158E substitutions.

To determine if GBV-C NS5A expression was specific for HIV replication inhibition, Jurkat cells stably expressing the full-length GBV-C NS5A (1–414), the 152–181 NS5A peptide, vector control (expressing GFP), and parental Jurkat cells were infected with mumps virus (MOI = 1.0; Jeryl-Lynn [vaccine] strain). The infectious titer of mumps in culture supernatants four days post-infection in Vero cells demonstrated that mumps replicated equally well in the parent, vector control, and the NS5A (152–181) Jurkat cell lines (Fig. 5A). Interestingly, the mumps titer was reproducibly higher in cells expressing full-length GBV-C NS5A (1–414)(p = 0.029), indicating that GBV-C NS5A somehow enhanced mumps virus replication (Fig. 4A). Since GBV-C NS5A 152–181 and 1–414 expression inhibited HIV replication, the enhancement in mumps replication appears to be mediated by a different region within NS5A. To further assess the specificity of the HIV inhibition, VSV-G pseudotyped HIV particles containing a luciferase reporter gene were prepared and assessed for their ability to transduce the various cell lines. Non-enveloped HIV particles containing the luciferase reporter served as the negative control particles. Figure 5B demonstrates that VSV-G transduction efficiency was not statistically different in any of the NS5A expressing cells compared to the vector control, irrespective of their HIV inhibitory phenotype.

### GBV-C NS5A peptide expression and chemokine receptors

**Figure 6:**
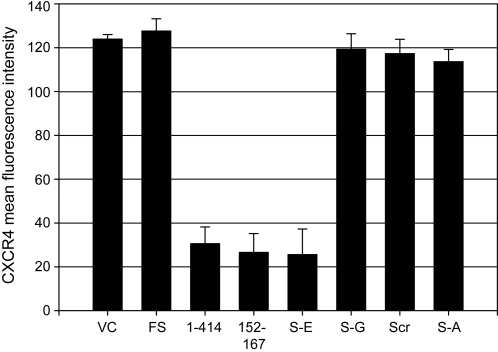
GBV-C NS5A peptides downregulate CXCR4 expression. Cells expressing the full-length NS5A (1–414), the 152–167 and S158E peptide had significantly less CXCR4 on their surface than did cells containing the vector control (VC), frameshift (FS), scrambled 152–167 peptide sequence (Scr), or the S158G, and S158A peptides. Data represent the mean fluorescent intensity of CXCR4 on the surface of these cells measured by flow cytometry.

Expression of GBV-C NS5A in Jurkat cells was previously shown to downregulate CXCR4 surface expression [33]. Jurkat cell lines were examined by flow cytometry to determine if the GBV-C NS5A peptides altered surface expression of HIV co-receptors CXCR4 and CCR5. CXCR4 was reduced on cells expressing the GBV-C NS5A peptides in which HIV replication was most potently inhibited, but not in control cell lines or in cell lines expressing the S158A, S158G, or scrambled peptides (Fig. 6). CCR5 was not detected on any of the Jurkat cell lines including the parent cell line (data not shown).

**Figure 7:**
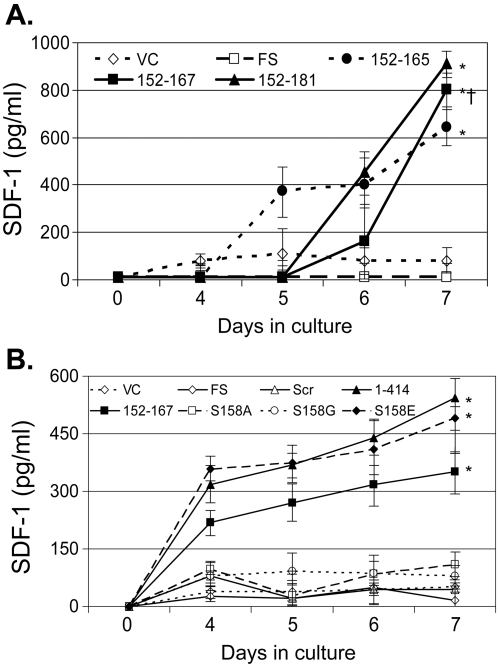
GBV-C NS5A peptides increase the release of stromal cell-derived factor 1 (SDF-1). SDF-1 release into culture supernatants was significantly greater in cell lines expressing GBV-C NS5A peptides 152–165, 152–167 and 152–181 compared to the vector control (VC) or frame shift (FS) cell lines after 7 days in culture (* p<0.01 for all; panel A). Jurkat cells expressing full-length NS5A (1–414), the 152–167 peptide and the S158E mutant peptide also induced significantly more SDF-1 than did cells expressing VC, FS or the S158A and S158G peptides (B; * p<0.02 for all on day 7).

SDF-1 release into culture supernatants was induced in cells in which CXCR4 expression was diminished, including cell lines that express GBV-C NS5A amino acids 152–165, 152–167, and 152–181 (fig. 7A) compared to the vector and frame-shift controls (p<0.01 for all three). Consistent with these findings, cells expressing the full-length NS5A (1–414), 152–167 and the S158E phosphomimetic substitution induced SDF-1 release compared to the cells expressing the vector control, the scrambled peptide, S158A, and S158G peptides and the frame-shift sequences (Fig. 7B; p<0.02 for all). There was variability in the amount of SDF-1 released in different experiments; however, the pattern of induction was similar between experiments. Cell lines expressing the parental NS5A sequences including 152–165 released significantly more SDF-1 than the vector, frame-shift and scrambled peptide controls when maintained in culture. The S158A and S158G peptides did not induce SDF-1, even though HIV replication was reduced compared to controls (Fig. 7A). This provides additional evidence that SDF-1 induction is not the sole mechanism by which NS5A inhibits HIV replication in these cells. Previous studies demonstrated that addition of ≥350 pg/mL SDF-1 inhibited HIV replication in CD4+ cell culture [10], thus the amount of SDF-1 released into supernatants is sufficient to inhibit HIV replication. Of note, SDF-1 release did not increase until days 4 or 5, further indicating that this is not the sole mechanism of HIV inhibition. RANTES, MIP-1α and MIP-1β were not detected in any of the culture supernatants (data not shown).

### Synthetic GBV-C NS5A peptides inhibit HIV replication

**Figure 8:**
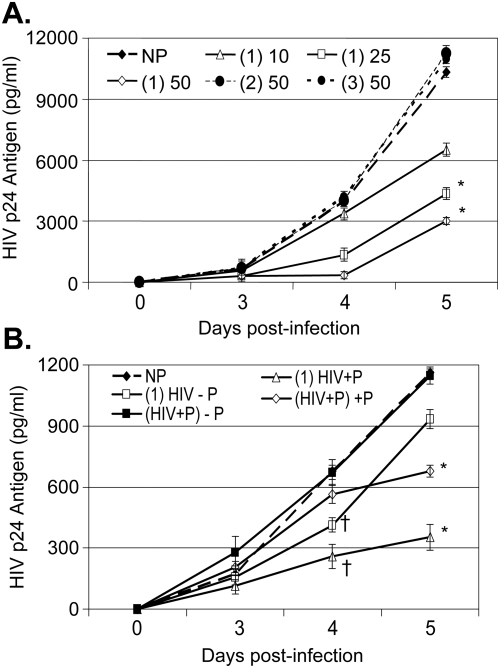
GBV-C NS5A synthetic peptides inhibit HIV replication. Addition of a synthetic peptide representing GBV-C NS5A amino acids 152–191 (P1) to MT-2 cells 1 hour before HIV infection resulted in a dose-dependent inhibition of HIV replication (concentration of peptide = 10, 25, or 50 µg/ml; panel A). By comparison, overlapping peptides including GBV-C NS5A amino acids 172 to 211 (P2) or amino acids 197 to 236 (P3) did not inhibit HIV replication at 50 µg/mL. All HIV replication was compared to cells incubated with no peptide (NP). Addition of peptide 1 (amino acids 152–191) to Jurkat cells 24 hrs prior to HIV infection [(1) HIV] resulted in inhibition of HIV on day 4 († p<0.05; panel B). In cells in which peptide was not included after HIV infection (− P), HIV inhibition was lost on day 5. When HIV and the peptides were added to cells simultaneously (HIV+P), inhibition was not observed on day 4; however, when the peptide was included in the media (+P), HIV replication was inhibited by day 5 (* p<0.05 compared to the NP control).

Based on our initial data demonstrating that an 85 amino acid fragment within NS5A inhibits HIV replication [33], overlapping peptides representing GBV-C NS5A amino acids 152–191, 172 to 211, and 197 to 236 were synthesized and tested for their ability to inhibit HIV when added to CD4+ T cell lines. To test the peptides in a cell line that supports high level HIV replication, MT-2 cells were incubated with the three peptides for 24 hrs prior to HIV infection and peptides were maintained in the media following HIV infection. The peptide representing GBV-C NS5A amino acids 152–191, but not the other two NS5A overlapping peptides inhibited HIV replication compared to the no peptide control on the fifth day post-infection in a dose-dependent manner (37% at 10 µg/mL; 58% at 25 µg/mL and 71% at 50 µg/mL; figure 8A). Although the magnitude of HIV inhibition was less than that observed when the peptides were expressed within Jurkat cells, the inhibition was statistically significant (p<0.01 at 25 and 50 µg/ml).

To further assess if the timing that peptides were added to cells, or if the type of CD4+ T cell line influenced the inhibitory effect, Jurkat cells were incubated with the inhibitory peptide (152–191) 24 hrs prior to HIV infection or at the time of HIV infection. Following HIV infection, cells were washed and incubated either with or without the peptide. As expected the magnitude of HIV replication was significantly lower in the control Jurkat cells compared to the control MT-2 cells (Fig. 8B). However, a similar pattern of HIV inhibition by the 152–191 NS5A peptide was observed in cells incubated with the peptide for 24 hrs prior to HIV infection compared to controls. This inhibition persisted for the first 4 days post infection, whether or not the peptide was maintained in the media (Fig. 8B). However, the inhibitory effect was lost by day 5 in cells maintained in media without peptide. In contrast, addition of peptides to cells at the time of HIV infection did not lead to significant differences in HIV replication from controls during the first 4 days post-infection. Nevertheless, cells maintained in peptide post-HIV infection demonstrated significantly less HIV release on day 5, presumably due to inhibition of cell-to-cell HIV spread (Fig. 8B).

**Figure 9:**
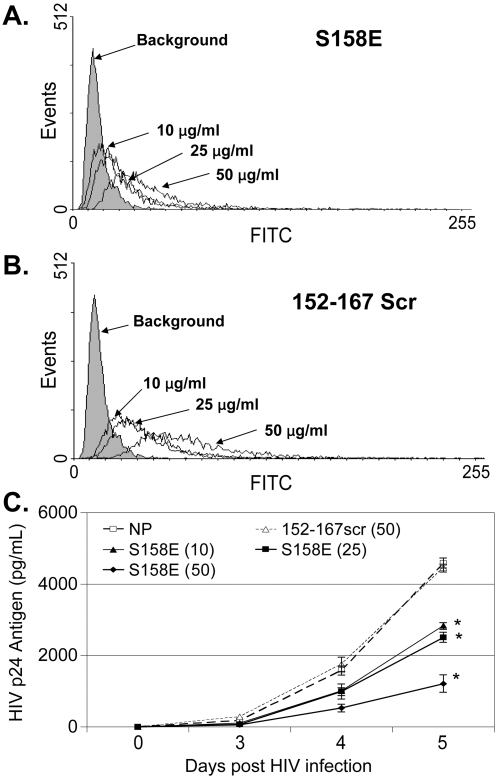
Dose dependent inhibition of HIV replication by the S158E mutant. MT-2 cells were incubated with three concentrations (10, 25, and 50 µg/ml) of synthetic peptides containing GBV-C S158E (A) or 152–167scr (B) amino acid sequences in which an N-terminal Tat-protein transduction domain was included, and the peptides were conjugated to FITC. Cells were washed one hour later and FITC was monitored by flow cytometry. Cells were infected with HIV 24 hrs after incubation, and dose related inhibition of HIV replication was observed for the S158E peptide, but not the 152–167scr peptide compared to the no peptide control (NP). * HIV p24 antigen area under the curve was less than controls (p<0.01).

Based on the deletion mapping and mutagenesis data (Figures 1 and 3), the active NS5A peptide mutant (S158E) and a control (152–167scr) peptide were synthesized and tested for HIV inhibition when added to cells 24 hrs prior to HIV infection (concentration up to 50 µg/mL). Neither of these peptides significantly inhibited HIV replication (data not shown). To determine if this reflected poor uptake and/or degradation of the peptides, the S158E peptide and the scrambled 152–167 peptide were synthesized with an N-terminal Tat-protein-transduction domain to improve cellular uptake [39], and the peptides were conjugated to FITC to allow monitoring of cellular binding and uptake. Both the Tat-S158E and Tat-152–167-scrambled fusion peptides were taken up by MT-2 cells in a dose-dependent manner (Fig. 9A and B). Cells incubated in the S158E peptide inhibited HIV replication compared to the no peptide control (Fig. 9C), albeit to a lesser extent than expression of the peptide within Jurkat cells (Fig. 3). In contrast, the Tat-152–167scr fusion peptide did not inhibit HIV (Fig. 9C), even though it's cellular uptake was similar to the S158E peptide (Fig. 9A and B). No cellular toxicity or effect on viability was observed in MT-2 cells or Jurkat cells incubated in either of the peptides (50 ug/ml) as determined by cell counts, trypan blue exclusion, and MTT assay [9], [25], [40] (data not shown).

## Discussion

The protein requirement within the GBV-C NS5A protein that is responsible for inhibiting HIV infection *in vitro* was further characterized in this study. Expression of ten different NS5A polypeptides containing amino acids 152–165 inhibited HIV in Jurkat cells (Fig. 1); however, scrambling the amino acid order of the 152–167 peptide abolished the inhibitory effect. Furthermore, the serine residue at position 158 appears important for the HIV inhibitory function (Fig. 3B), since a phosphomimetic substitution (S158E) inhibited HIV as well as the expression of either the full length NS5A protein or the parent peptide. Of note, the parent peptide and S158E peptide inhibited HIV significantly more than the S158A and S158G peptides; however, these peptides also inhibited HIV replication compared to the scrambled peptide, frame-shift and vector controls (Fig. 3C, 4A and 4B). These data are consistent with the hypothesis that the serine at position 158 is important for the HIV inhibitory effect of this peptide, and suggest that either phosphorylation or a structural motif of the peptide is required for HIV inhibition. The GBV-C NS5A inhibition effect was specific for HIV, as both mumps virus replication and the transduction efficiency of VSV-G pseudotyped HIV particles was not diminished in the NS5A expressing cells in which HIV replication was inhibited. Interestingly, full-length NS5A (1–414) enhanced mumps replication in Jurkat cells compared to parental and vector control cells. To our knowledge, viral enhancement has not been previously described for flavivirus NS5 proteins. However, expression of HCV NS5A in a HepG2 cell line was shown to enhance hepatitis B protein expression and virus replication, potentially as a result of NS5A partial inhibition of interferon responsiveness [41]. Ongoing studies are examining potential mechanisms of the mumps virus replication enhancement by GBV-C NS5A. Nevertheless, since the GBV-C 152–181 protein did not enhance mumps virus replication, the HIV inhibitory domain within GBV-C NS5A is distinct from the mumps-enhancing domain. This enhancement in mumps replication further emphasizes the data showing that NS5A expression is not toxic to the Jurkat cells.

Although GBV-C NS5A protein function is not completely understood; in the closely related HCV, NS5A is required for viral replication and is part of the viral replication complex [42]–[44]. NS5A expression is confined to actively infected cells, which comprise fewer than 5% of PBMCs *in vitro* [25], [26] and an unknown percent *in vivo*. Thus it is unlikely that the effect of GBV-C NS5A expression is directly responsible for all of the effect of GBV-C on HIV replication and disease progression. However, because GBV-C NS5A and the NS5A peptides downregulate the HIV co-receptor CXCR4 and induce the release of SDF-1, NS5A protein expression would appear to have a bystander effect on cells not infected with GBV-C. Consistent with these findings, previous studies demonstrated that GBV-C infection of healthy PBMCs *in vitro* resulted in the induction of SDF-1 [10], [29], and prior studies with full-length NS5A expressing Jurkat cells found that neutralizing SDF-1 activity with neutralizing antibodies diminished but did not abolish the HIV inhibitory effect [33]. Thus, additional mechanisms of HIV inhibition appear to be elicited by the GBV-C NS5A protein.

Addition of a synthetic peptide representing GBV-C NS5A 152–191 amino acids and the S158E mutant peptide to Jurkat and MT-2 cells reproducibly inhibited HIV replication, although to a less extent than that observed when the peptides were expressed intracellularly in Jurkat cells (Fig. 2, 3, 7, and 8). The S158E peptide was not active by itself; however, the addition of a tat-protein transduction domain to the amino terminus resulted in HIV inhibition for the S158E peptide but not the 152–167scr peptide. These data suggest, but do not prove that intracellular uptake of the native peptide and/or degradation may have contributed to it's lack of efficiency.

The secondary structure of the 152–167 peptide and S158E mutant is predicted to contain a beta-strand (DNAMan software, Lynnon Biosoft). In contrast, the S158A, S158G, and 152–167scr peptides are not predicted to form a beta strands, suggesting that a critical peptidic structure is responsible for the HIV inhibitory interaction. The activity of the S158E peptide appeared to be potentially greater than the parent peptide (Fig. 4B). Comparison of the probability of the predicted structures indicates that the probability of a beta-strand is greater in the S158E peptide than the parent peptide, suggesting that this substitution may result in a more stable structure than the parent sequence. Alignments of the GBV-C NS5A 152–167 amino acid sequence with NS5 sequences of isolates representing related flaviviruses (HCV, Dengue virus, yellow fever virus, and West Nile Virus) did not identify >32% homology. In addition, Blast search (http://www.ncbi.nlm.nih.gov/blast/Blast.cgi) of all proteins (non-redundant GenBank CDS translations+PDB+SwissProt+PIR+PRF excluding environmental samples from WGS projects) did not identify proteins sharing homology with the 152–167 peptide or the S158E peptide.

GBV-C viremia is associated with prolonged survival in HIV-infected people in most studies conducted prior to the availability of combination antiretroviral therapy [4], although some argue that GBV-C is a marker of low HIV RNA [45] or high CD4 counts [46], [47]. Demonstration that NS5A peptide expression inhibits HIV replication adds to previous *in vitro* studies that support a causal relationship between GBV-C viremia and survival in HIV-infected individuals [4]. Like HIV, GBV-C replicates in CD4+ lymphocytes and in addition, GBV-C replicates in CD8+ T cells and B cells [26]. Since the NS5A 152–167 peptide induces the release of SDF-1 and downregulates CXCR4 surface expression (figure 5, 6 and reference [33]), it is reasonable to speculate that the bystander effect of NS5A expression and chemokine modulation would influence HIV replication in lymphoid tissue. Further studies to characterize the structural requirements of the GBV-C 152–167 NS5A peptide, and to identify intracellular interacting proteins are underway. These data suggest that the GBV-C NS5A peptide may be useful from a therapeutic standpoint. Because the peptide functions by altering CXCR4 and inducing SDF-1 instead of inhibiting HIV replicative enzymes, it may provide a therapeutic approach that is more difficult for HIV to overcome by the selection of virus mutations.

## Materials and Methods

### GBV-C NS5A proteins and peptides

The full length GBV-C NS5A protein coding sequence (numbering based on AF121950) was previously amplified from the plasma of an individual with GBV-C viremia, ligated into the pTRE2-Hyg plasmid (Clontech, Inc., Mountain View, CA) modified to include a stop codon after NS5A, followed by the encephalomyocarditis virus (EMC) IRES element directing the translation of green fluorescent protein (GFP) as described [33]. GBV-C NS5A deletion mutants were generated by using convenient restriction sites as previously described [33], and all of the peptide constructs were generated by ligating synthetic oligonucleotides containing flanking NheI and NotI restriction sites into the modified pTRE2-Hgy plasmid [33]. All sequences were confirmed by automated fluorescent dye terminator cycle sequencing (University of Iowa DNA Core Facility; Applied Biosystems automated DNA sequencer 373A, Foster City, CA). Tet-Off Jurkat cells (Clontech, Inc.) were transfected (Amaxa nucleofection, Amaxa Inc., Gaithersburg, MD) with plasmids containing NS5A sequences or with control sequences including the vector control expressing GFP, or with a vector that contains NS5A sequences with a single base insertion to create a frameshift mutation as previously described [33]. Following selection in hygromycin and neomycin (200 µg/ml each), bulk cell lines were examined for GFP expression. Cell lines stably expressing GFP were examined for support of HIV replication and CXCR4 surface expression, and clonal cell lines were prepared by at least two rounds of terminal dilution cloning.

To monitor NS5A expression, Jurkat cells were lysed in RIPA buffer containing protease and phosphatase inhibitors, clarified (13,000× g, 2 min at 4oC), and subjected to SDS-PAGE prior to transfer to nitrocellulose membranes (Bio Rad, Inc., Hercules, CA) as previously described [36]. Immunoreactive proteins were identified using the GE3 anti-GBV-C NS5A rabbit serum kindly provided by Dr. Jungsuh Kim (Genelabs Technologies, Inc., Redwood City, CA) which was generated against GBV-C nucleotide sequences 6615–6977 expressed in E.coli. For cell lines expressing NS5A fragments not detected by immunoblot, expression of GFP was demonstrated by flow cytometry and total cellular DNA and RNA was examined for linkage between NS5A sequences and GFP using PCR or RT-PCR followed by determination of the nucleotide sequence as previously described [33], [35]. DNA sequence alignments and predicted protein secondary structure analyses employed DNAMan software (Lynnen Biosoft, Inc., Pointe-Claire, Quebec, Canada).

Synthetic peptides representing NS5A amino acid sequences were either kindly provided by Dr. Opendra Sharma and the NIH Aids Reference Reagent Program or purchased (New England Peptide LLC, Gardener MA or Iowa State University Protein Facility, Ames, IA).

### Virus infections

An HIV-1 isolate (X4, clade B; NIH AIDS Research and Reference Reagent Program, catalog number 1073) was used to infect CD4+ T cell lines (Jurkat cells and Jurkat cells expressing GBV-C NS5A peptides or control plasmids, or MT-2 cells; 200 pg HIV p24 antigen per 10^6^ cells) as previously described [48]. Following HIV attachment, cells were washed, maintained in fresh media and culture supernatants were obtained at various time points to measure HIV-1 replication. HIV-1 replication was determined by measuring HIV p24 antigen in culture supernatant fluids (Retro-Tek HIV-1 p24 antigen ELISA kits, Zeptometrix, Buffalo, NY) as previously described [9], [10]. All infections were performed in triplicate and were independently repeated at least twice with consistent results.

Attenuated mumps virus (Jeryl-Lyn vaccine strain, Merck & Co., Whitehouse Station, NJ) was purchased and a stock virus preparation was generated to serve as a specificity control. The infectious titer of this preparation was determined in Vero cells as described by others [49]. The mumps virus preparation was used to infect Jurkat cells including cell lines that expressed GBV-C NS5A peptides or vector control cell lines.

Pseudotyped HIV particles were generated by transient transfection of plasmid DNA into 293T cells plated 2 days prior to transfection at a density of 1×10^6^ per 10-cm-diameter culture dish. Two plasmid co-transfections were performed using 10 µg of HIV packaging construct (pNL4-3,Luc.R-E-; NIH AIDS Research and Reference Reagent Program catalog # 3417) and 10 µg VSV-G envelope-expressing plasmid (pHEF-VSVG; NIH AIDS Research and Reference Reagent Program catalog #4693)[50]. HIV particles without an envelope served as the control. DNA complexes were prepared with calcium chloride and transfected into cells as described [50], and medium was replaced 4 h after transfection. Culture supernatants were harvested 72 hours after the start of transfection, filtered through a 0.45 µm Nalgene filter, and stored at 4°C prior to use. Jurkat cells (8×10^4^ cells/well) were placed into a 24-well plate and transduced with VSV-G pseudotyped HIV or the no-envelope HIV (gag) particles. Cells were lysed with Glo-Lysis buffer (Promega, Inc., Madison, WI) 72 hours after transduction and relative luciferase activity assessed as recommended by the manufacturer on a TD-20/20 Luminometer (Turner Biosystems, Sunnyvale, CA).

### Chemokines and chemokine receptors

CXCR4 and CCR5 expression on the surface of cells were determined by flow cytometry as previously described [9]. Polyclonal rabbit anti-CCR5 (FITC-conjugated) and anti-CXCR4 (PE-conjugated) antibodies (BD Pharmingen, San Jose, CA) were used in these studies. Flow cytometry was performed using a FACScan (Becton Dickenson, San Jose, CA). SDF-1, RANTES, MIP-1α and MIP-1β were detected in replicate culture supernatant fluids by ELISA (R&D Systems, Minneapolis, MN) as previously described [10].

### Statistics

Statistics were performed using SigmaStat software V3.11 (Jandel Scientific, Chicago, IL). HIV replication was analyzed by measuring HIV p24 antigen release or infectious titer (mumps virus) on various days post-infection, and comparisons were performed using T-tests.
